# Identical Viral Genetic Sequence Found in Black Flies (*Simulium* *bivittatum*) and the Equine Index Case of the 2006 U.S. Vesicular Stomatitis Outbreak

**DOI:** 10.3390/pathogens10080929

**Published:** 2021-07-23

**Authors:** Barbara S. Drolet, Will K. Reeves, Kristine E. Bennett, Steven J. Pauszek, Miranda R. Bertram, Luis L. Rodriguez

**Affiliations:** 1 Arthropod-Borne Animal Diseases Research Unit, Center for Grain and Animal Health Research, Agricultural Research Service, Unites States Department of Agriculture, Manhattan, KS 66502, USA; 2 Biological Regulatory Services, Animal and Plant Health Inspection Service, United States Department of Agriculture, Fort Collins, CO 80526, USA; will.k.reeves@usda.gov; 3 Energy Institute, Colorado State University, Fort Collins, CO 80523, USA; kristine.bennett@colostate.edu; 4 Foreign Animal Disease Diagnostic Laboratory, Plum Island Animal Disease Center, National Veterinary Services Laboratories, Animal and Plant Health Inspection Service, United States Department of Agriculture, Orient Point, NY 11957, USA; steve.pauszek@usda.gov; 5 Foreign Animal Disease Research Unit, Plum Island Animal Disease Center, Agricultural Research Service, Unites States Department of Agriculture, Orient Point, NY 11957, USA; miranda.bertram@usda.gov (M.R.B.); luis.rodriguez@usda.gov (L.L.R.)

**Keywords:** vesicular stomatitis virus, horse, black fly, *Simulium bivittatum*

## Abstract

In 2006, vesicular stomatitis New Jersey virus (VSNJV) caused outbreaks in Wyoming (WY) horses and cattle after overwintering in 2004 and 2005. Within two weeks of the outbreak onset, 12,203 biting flies and 194 grasshoppers were collected near three equine-positive premises in Natrona County, WY. Insects were identified to the species level and tested by RT-qPCR for VSNJV polymerase (L) and phosphoprotein (P) gene RNA. Collected dipterans known to be competent for VSV transmission included *Simulium* black flies and *Culicoides* biting midges. VSNJV L and P RNA was detected in two pools of female *Simulium bivittatum* and subjected to partial genome sequencing. Phylogenetic analysis based on the hypervariable region of the P gene from black flies showed 100% identity to the isolate obtained from the index horse case on the same premises. This is the first report of VSNJV in *S. bivittatum* in WY and the first field evidence of possible VSV maintenance in black fly populations during an outbreak.

## 1. Introduction

Vesicular stomatitis (VS) is a vector-borne, zoonotic disease caused by the RNA virus vesicular stomatitis virus (VSV), a *Vesiculovirus* in the family *Rhabdoviridae*, which primarily affects horses, cattle, and swine with clinical signs that include transient fever, excessive salivation, and lesions in the oral cavity, on the lips, nose, coronary bands, and teats of livestock [1,2]. Because these lesions in cattle and swine resemble those caused by foot and mouth disease virus, VS is designated as a reportable disease resulting in economic losses primarily due to animal quarantines and trade restrictions [3,4]. Incursions of VSV into the U.S. from endemic regions in southern Mexico occur sporadically at 5–10-year intervals, resulting in single or multi-year outbreaks, presumably by overwintering through an as yet unknown mechanism [3].

Active pathogen-based annual surveillance of VS in the U.S. is limited. Accredited veterinarians are required to immediately report cases of suspected vesicular lesions to state and federal animal health officials. All livestock with reported lesions are tested for VSV as part of the vesicular diseases rule-out process. During years where VS does not occur, the U.S. Department of Agriculture (USDA), Animal and Plant Health Inspection Service (APHIS) typically tests 150–200 premises nationwide in response to reported lesions that are determined to be something other than VS. In years when VS outbreaks have been confirmed, reports of suspected vesicular lesions increase significantly, with USDA, APHIS testing 800–1000 premises nationwide, although a proportion of those reports are determined diagnostically to be something other than VS. The only VS testing performed on non-clinical livestock is for the purpose of export to other countries, which varies widely from year to year and depends on the testing requirements of the receiving country. The use of seroprevalence testing to determine outbreak status or transmission rates is of limited value due to persisting antibodies in exposed animals. Specifically, previous exposure of horses during a single VS outbreak year resulted in VS-positive horses maintaining antibody titers for 10–12 years [5,6]. For these reasons, USDA policy is to only perform VS testing on non-clinical animals if required for export. Thus, the first report of lesions, with positive confirmatory PCR swab testing, is reported as the first VS-positive premises for a specific region or state. Confirmed positive and suspect premises are quarantined for at least 14 days from the onset of lesions in the last affected animal on the premises [7].

During outbreaks, viral transmission can occur within herds by direct contact between infected animals, which shed copious amounts of virus in lesions and saliva, or by fomites [2,8]. Several insect species have been incriminated as both mechanical and biological vectors which acquire VSV from blood, lesions, or saliva [9,10,11]. They are believed to play an important role in the transmission of virus within and between herds, especially when quarantines and stop-movement measures are in place [11,12,13,14,15,16,17,18,19,20]. During U.S. outbreaks, VSV has been isolated from several Dipteran species including mosquitoes (*Aedes* spp.) [21], biting midges (*Culicoides* spp.) [22,23], black flies (*Simulium* spp.) [10,20], and from sand flies (*Lutzomyia shannoni)* on Ossabaw Island, GA where VS was endemic until feral swine populations were removed [12,24,25,26]. Grasshoppers (*Melanoplus* spp.) were shown in laboratory settings to become infected by eating VSV-contaminated pasture plants [27], amplify the virus, and transmit it to cattle that ingest them [15]; however, there are no reports of field-collected grasshoppers during outbreaks.

In addition to field evidence for vector incrimination, experimental studies with colonized and wild-caught *Simulium* black flies have demonstrated VS New Jersey virus (VSNJV) replication [13,28], VS Indiana virus (VSINV) replication [29], and bite transmission of VSNJV [14,30,31]. *Simulium bivittatum*, a common black fly species across the western U.S., produces several generations over the vector season. Eggs, larvae, and pupae develop in flowing fresh water; only the adult stage is terrestrial [32,33]. Adults emerge and immediately seek food sources and mates, dispersing less than 5 km from their origin at first, but females can disperse hundreds of km during their 10- to 35-day average lifespan to find hosts for blood meals and suitable habitat for oviposition [32]. Many *Simulium* spp., including *S. bivittatum*, feed on livestock and large animals in the ears, on other sparsely-haired areas of the head, and on the underbelly [32]. They are significant pests of horses [34,35].

A three-year U.S. VS outbreak in horses and cattle occurred between 2004 (NM, TX, CO; 294 premises in 43 counties), 2005 (NM, AZ, TX, UT, CO, WY, NE, MT, ID; 445 premises in 69 counties), and 2006 (WY; 13 premises in 3 counties) [36,37,38]. State-imposed quarantines were lifted in WY on 28 December, 2005 [39]. The index case for 2006 was a horse with clinical lesions reported nine months later on 13 August [38,39] in Natrona County, WY, with 29 clinically normal horses and 25 clinically normal cattle also on the premises. Sequencing and phylogenetic analysis revealed a shared genotype from virus isolated in 2004 and 2005 [40], and a second shared genotype from virus collected in 2005 and 2006 [38]. Phylogeographic data support the virus overwintering in NM, TX, and CO from 2004–2005 [40] and in WY from 2005–2006 [39].

In the absence of clinically infected livestock between multiyear outbreak seasons, and with no known mammalian wildlife reservoir for VSV, insects have been suggested as likely overwintering reservoirs [12,41,42,43]. To examine the insect species possibly involved in maintaining the outbreak and potential insect sources for overwintering of the virus in WY between the last resolved case in December 2005 and the index case in August of 2006, biting flies were caught in light traps placed on the outbreak premises 11 days after the first clinical case was reported. Grasshoppers near traps were also collected by hand netting. Collections were in late August, less than a month prior to first freeze dates in Natrona County, WY (www.crh.noaa.gov/riw/climate/clm/lcdcpr.php accessed on 3 June 2021). Insects collected were likely the last generation for the year and would have either overwintered as adults or laid potentially infected eggs. All biting flies and grasshoppers were tested for VSNJV by RT-qPCR and virus isolation in cell culture, and sequencing was attempted for positive samples.

## 2. Results

### 2.1. Collection Sites

Information on the geographic location (latitude, longitude) of the VSNJV-infected premises was provided by USDA, APHIS. The 2006 VSNJV outbreak in the U.S. started in Natrona County, WY with the first reported lesion in a horse on 13 August. Swabs of lesions and blood samples were obtained by the attending veterinarian and submitted to the USDA, APHIS, National Veterinary Services Laboratories (NVSL; Ames, IA). VSNJV was confirmed in lesion swabs by virus isolation and VSV antibody was confirmed by cELISA. The VS-compatible clinical signs and the presence of virus met the definition to classify this horse as the index case for the nation. The isolate was obtained and the whole genome was sequenced at the USDA, Agricultural Research Service (ARS), Plum Island Animal Disease Center (PIADC) in Orient Point, NY (GenBank, Dec. 2020, MT094085.1) [44]. During the 2005 outbreak, Natrona County reported only two cases, with lesion onset dates of 30 October and 6 November. Thus, the last infected premises for WY occurred nine months earlier and was located four miles west of the first case in 2006. By 20 August, three positive premises, of what would ultimately be a total of eight in the county, had been confirmed. Permissions were received from property owners of the three premises for insect collection efforts, which began on 23 August.

### 2.2. Detection of VSV RNA in Insect Collections

A total of 12,203 biting flies and 193 grasshoppers were collected from three premises in one county of WY during the VS outbreak and sorted by species. Biting fly species collected included *Aedes*, *Culex,* and *Culiseta* mosquitoes, *Stomoxys calcitrans* and *Hematobia irritans* biting flies, *Culicoides* biting midges, and *Simulium* black flies (Table 1). Additionally, grasshopper species collected included *Melanoplus gladstoni*, *M. femurrubrum,* and *M. sanguinipes*, *Spharagemoh collare*, *Opeia* spp., *Chorthippus curtipennis*, and *Paropomala wyomingensis*. No VSNJV RNA was detected by RT-qPCR in any of the mosquitoes, the *Stomoxys calcitrans* or *Hematobia irritans* biting flies, the *Culicoides* biting midges (*C. sonorensis* and *C. steliffer*), nor in any of the grasshoppers.

Four *Simulium* black fly species were collected: *S. arcticum, S. bivittatum, S. griseum,* and *S. tribulatum*. Of these, two pools of *S. bivittatum* from one trap set in the east Casper pasture near the North Platte River were positive for both the polymerase (L) and phosphoprotein (P) genes of VSNJV (Table 1). Flies did not have a blood meal at the time of collection so viral RNA detection indicates an active infection in the insect, as opposed to detection of virus from an infectious meal in the gut. The trap was located near an outdoor hay bailer in a large pasture that held six horses with no vesicular lesions. This pasture was located approximately 200 yards from an enclosure where the clinically infected horse was housed. The premises was four miles east of the last positive premises in 2005. The lesion onset date was reported to APHIS as 13 August 2006 (11 days prior to insect collections), at which time a vesicle swab was sent to USDA, APHIS, NVSL for diagnostic evaluation of a suspected VSV infection and eventually sent to PIADC for phylogenetic analyses. Virus isolation attempts with the remaining original RT-qPCR-positive black fly homogenate samples by passage in Vero cell cultures were unsuccessful.

### 2.3. Sequencing and Phylogenetic Analysis of VSNJV-Positive Black Flies

**Figure 1:**
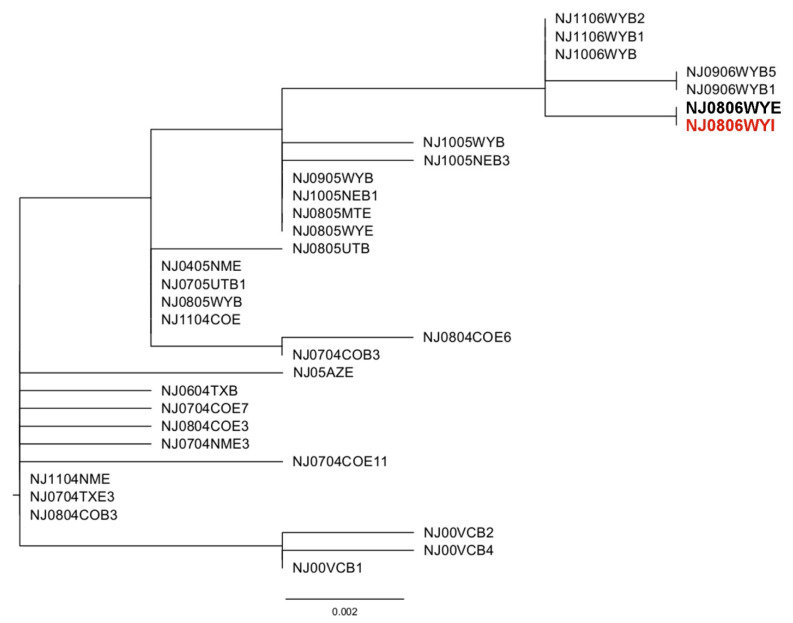
Maximum-likelihood analysis of vesicular stomatitis New Jersey virus (VSNJV) isolates from the 2004–2006 U.S. outbreak deduced from the phosphoprotein gene hypervariable region. Tree was rooted with a closely related viral lineage from an endemic region in Mexico (Veracruz). Metadata is contained in each sequence name: serotype (NJ: New Jersey), month (when available), year, state (two letter code for U.S. states, VC: Veracruz, Mexico), host (B: bovine, E: equine, I: insect), and isolate number (if more than one with the same name). Representative sequences of each previously identified distinct strain are included in the analysis. The 2006 black fly VSNJV isolate NJ0806WYI (bold red), collected 24–25 August, is 100% identical to NJ0806WYE (bold), collected 13 August from the equine index case on the same premises.

Complete sequence coverage of the 450 nt hypervariable region of the P gene was obtained from one of the two RT-qPCR VSV-positive *S. bivittatum* pools (NJ0806WYI). Phylogenetic analysis with sequences from the 2004–2006 U.S. outbreak showed the NJ0806WYI black fly sequence formed a monophyletic clade with the other six sequences collected in 2006 and was closely related to sequences collected in 2005 in the same region (Figure 1). The NJ0806WYI black fly pool, collected on 24–25 August 2006, was 100% identical across the 450 nt hypervariable region to NJ0806WYE (GenBank MT094085.1), the isolate recovered from the equine index case sample collected on 13 August 2006 from the same premises. It was 99.3% identical (3 nt differences over the 450 nt sequence) to two 2005 Wyoming isolates: a horse (NJ0805WYE) and a cow (NJ0905WYB) from the previous year’s outbreak. Further phylogenetic characterizations of the 2004–2006 U.S. outbreak were reported previously [38,40].

## 3. Discussion

A diverse number of arthropod species have been identified as confirmed or suspected vector species for VSV [11]. Both *S. vittatum* and *S. notatum* have been demonstrated as vectors of VSV under laboratory conditions [13,29,45]. Our data support the previous evidence of *Simulium* spp. as vectors of VSNJV. In addition, VSNJV has been isolated from *S. bivittatum, S. vittatum/S. tribulatum,* and undetermined *Simulium* (*Psilopelmia*) spp. from VS outbreaks in Colorado [10,20]. Additional incriminated vectors in the U.S. include *C. sonorensis* biting midges [16,17,18,19] and *Lutzomyia shannoni* sand flies [26]. Suspected vectors include *C. stellifer* [23] and migratory grasshoppers [15,27]. These insects were identified in the collections, but none were positive for VSNJV.

We detected VSNJV L and P gene RNA by RT-qPCR from two black fly pools containing 13 and 20 *S. bivittatum*, consistent with previous studies implicating *Simulium* spp. as vectors of VSV [20]. Not surprisingly, the largest number of black flies were caught in traps on the two sites near running water (North Platte River and a spring-fed creek) providing a suitable larval fly habitat. *S. bivittatum* is in the subgenus *Psilopelmia,* which is the only subgenera of *Simuliidae* in North America that is still in need of taxonomic revision [32]. A better understanding of the distribution, biology, and taxonomy of species in the subgenus *Psilopelmia* could be critical to understanding the transmission and epidemiology of VS.

The overwintering and immature development of *S. bivittatum* in WY remains unstudied but should range between the known behaviors of individuals from Alberta, Canada, and Nebraska. In Canada, both larvae and pupae are found as late as September with an overwintering stage as eggs [46], and in Nebraska overwintering occurs as eggs with at least three generations per year from early April to late November [47]. Based on limited data from Utah, females of *S. bivittatum* lay around 200 eggs [48]. If virus overwintering occurs in flies, it is possibly from eggs laid in September, October, or later that hatch as larvae in the spring.

The P hypervariable sequence obtained from one black fly pool was 100% identical to virus isolated from the clinically infected equine index case on the same premises (NJ0806WYE) [38] and 99.3% identical to two 2005 Wyoming isolates (NJ0805WYE and NJ0905WYB). The potential role of *S. bivittatum* in the overwintering of VSV from 2005 to 2006 remains unresolved as this collection was 11–12 days after lesions were reported at the premises, and spillover infection from a natural reservoir cannot be ruled out. However, black flies were clearly involved early in the outbreak with the overwintering genotype, as the sequence was identical to the first positive premises reported for the U.S. Neither positive pool contained engorged nor gravid females, which suggests active feeding of these flies on the infected horse occurred 2 to 6 days prior with a subsequent gonotrophic cycle and establishment of a productive infection prior to collection. This is the first report of VSV-positive *S. bivittatum* during an outbreak in WY and the first field evidence of possible VSV maintenance in black fly populations during an outbreak. Where and how the virus persisted in WY for nine months between the last 2005 case and the first 2006 case, in the absence of clinically infected animals, remains undetermined.

## 4. Materials and Methods

*Insect collections*. Insects were collected between 23–25 August on three premises with active VS horse cases in Natrona County near Casper, WY (Table 1). Biting flies were trapped at dusk using CDC light traps baited with dry ice. In a pasture site in east Casper near the North Platte River, proximal to the first confirmed VSV-positive horse with a lesion onset date of 13 Aug, six traps were set for two nights. In a field site near an artesian spring south of Casper, proximal to one confirmed positive horse with a lesion onset date of 15 Aug, two traps were set for one night and one trap was used for two additional nights. At a farm site southeast of Casper near a spring-fed creek, with one positive horse with a lesion onset date of 20 August, three traps were set for two nights. Trap positions were selected to include locations near infected animals, near feeding and drinking areas used by infected and uninfected animals, and near potential insect breeding sites. Traps were retrieved at dawn and the insects were killed with CO_2_. Insects were sorted and pooled by taxon (1–50 flies per pool) (Table 1) on a chill table. Biting flies were identified using morphological characters [32,49,50]. Grasshoppers were hand netted from pastures at the three locations, killed by freezing, sorted by morphological characters [51], and processed individually.

*Real time RT-qPCR.* Each biting fly pool was homogenized in 500 µL of homogenization buffer (20% fetal bovine serum, 50 μg/mL streptomycin, 50 U/mL penicillin, and 2.5 μg/mL amphotericin B in phosphate-buffered saline) [52] with gold-plated tungsten beads using a Tissue Lyser (Qiagen, Germantown, MD, USA) as previously described [53]. Total RNA was extracted from 400 µL of each biting fly homogenate using an RNeasy kit (Qiagen) as per manufacturer’s instruction and stored at −80 °C. Individual grasshopper abdomens were homogenized in 1 mL of buffer [15] and total RNA was extracted from 900 µL of the homogenate as above. All RNA extracts were initially screened for VSV using RT-qPCR for detection of the L gene as previously described [54]. Positive pool RNA was subsequently tested for the most variable region of the virus, the P gene, as previously described [55].

*Virus isolations*. Isolation of infectious VSV from RT-qPCR-positive pools was attempted by placing 60 µL of the remaining 100 µL insect homogenates in 600 µL of antibiotic media (400 U/mL penicillin, 400 µg/mL streptomycin, 200 µg/mL gentamicin, 25 µg/mL ciprofloxacin (Serologicals, Inc, Norcross, GA, USA), and 5 µg/mL amphotericin B prepared in Medium 199 with Earle’s salts (M199-E; Sigma, St. Louis, MO, USA) in 10% fetal bovine serum) [43]. An inoculum of 200 µL per well (in triplicate) of a 6-well plate was added to Vero MARU (VM; Middle America Research Unit, Panama) cell monolayers with 2 mL M199-E (Sigma). Plates were incubated at 37 °C with 5% CO_2_ and checked microscopically for CPE daily. After 7 days, plates were freeze-thawed twice, and samples were passed onto fresh VM cell monolayers. Cells were checked daily for CPE as above and after 7 days, RNA was extracted and tested for VSV L gene by RT-qPCR.

*Sequencing*. The extracted total RNA of insect pools testing positive for VSV L and P gene RNA by RT-qPCR was reverse transcribed with random hexamers and shipped to PIADC for sequencing. Amplicon generation of the hypervariable region (450 nt) of the P gene was performed using previously described primers [40]. Amplicons were cloned using the Zero Blunt TOPO PCR cloning kit (Invitrogen, Carlsbad, CA, USA). Positive clones were processed with the QuickLyse Miniprep kit (Qiagen) and bi-directionally sequenced with the M13 forward and reverse primers included in the Zero Blunt kit using a BigDye Terminator Sequencing kit on a 3730A automated sequencer (Applied Biosystems, Thermo Fisher Scientific, Waltham, MA, USA). The consensus sequence for the positive pool was deduced from a total of 12 clones using Sequencher software v4.8 (GeneCodes, Ann Arbor, MI, USA).

*Phylogenetic analyses*. The P gene hypervariable region sequence recovered from the black fly pool was aligned with other publicly available sequences from the 2004–2006 U.S. outbreak [38]. Sequences from Mexican isolates collected in 2000 were included as an outgroup. Redundant sequences were removed, and sequences were aligned using Clustal W implemented in MegaX [56]. The Tamura 3-parameter model with invariant sites was identified as the most appropriate model, and a maximum likelihood phylogenetic tree was constructed using this model and 100 bootstrap replicates implemented in MegaX. The final consensus tree was visualized in FigTree v1.4.3 (tree.bio.ed.ac.uk/software/figtree).

## Figures and Tables

**Table 1:** Insect species collected from three sites in Natrona County, Wyoming in August of 2006.

Collection Site	Date	Identification	Number of Females	VSNJV PCR+ Pools/Total
East Casper pasture near North Platte River	24–25 August 2006			
		*Aedes dorsalis*	4789	0/96
		*Ae. melanimon*	7	0/1
		*Ae. nigramaculis*	11	0/1
		*Ae. vexans*	89	0/2
		*Culex tarsalis*	59	0/2
		*Culiseta inornata*	311	0/7
		*Culicoides crepuscularis*	21	0/1
		*C. sonorensis*	666	0/14
		*C. steliffer*	28	0/1
		*Hematobia irritans*	2	0/1
		*Simulium arcticum* complex	230	0/5
		***S. bivittatum***	**3108**	**2/63**
		*S. griseum*	590	0/12
		*S. tribulatum/S. vittatum*	2	0/1
South Casper field near artesian spring	23–25 August 2006			
		*Ae. dorsalis*	2	0/1
		*Cx. tarsalis*	22	0/1
		*C. sonorensis*	218	0/5
		*S. arcticum* complex	4	0/1
		*S. bivittatum*	84	0/2
		*S. griseum*	46	0/1
Southeast Casper Farm near creek	24–25 August 2006			
		*Ae. dorsalis*	859	0/18
		*Ae. melanimon*	303	0/7
		*Ae. nigramaculis*	50	0/1
		*Ae. vexans*	74	0/2
		*Cx. tarsalis*	196	0/4
		*Cs. inornata*	37	0/1
		*C. sonorensis*	4	0/1
		*H. irritans*	2	0/1
		*S. arcticum* complex	90	0/2
		*S. bivittatum*	33	0/1
		*S. griseum*	14	0/1
		*S. tribulatum/S. vittatum*	251	0/5
		*Stomoxys calcitrans*	1	0/1

Pools were tested for VSNJV polymerase and phosphoprotein gene RNA by RT-qPCR. Two pools of *Simulium bivittatum* were positive for both genes (bold).

## Data Availability

The data presented in this study are available upon request from the corresponding authors and through the USDA, Agricultural Research Information System.
